# Overtime Challenges of Diagnosis and Treatment in Two Pediatric Patients with Extensive Cerebral Tumefactive Lesions Indicative of Baló’s Type Multiple Sclerosis

**DOI:** 10.3390/children12050630

**Published:** 2025-05-14

**Authors:** Alice Denisa Dică, Dana Craiu, Catrinel Iliescu, Marcel-Alexandru Găină, Carmen Sandu, Cristina Pomeran, Carmen Burloiu, Alexandra-Maria Găină, Daniela Adriana Ion

**Affiliations:** 1 “Prof. Dr. Alexandru Obregia” Clinical Hospital of Psychiatry, 041914 Bucharest, Romania; alice.dica@drd.umfcd.ro (A.D.D.); dana.craiu@umfcd.ro (D.C.); catrinel.iliescu@umfcd.ro (C.I.); carmen.sandu@umfcd.ro (C.S.); cristina.pomeran@umfcd.ro (C.P.); carmenburloiu@gmail.com (C.B.); 2 Department of Pediatric Neurology, The Faculty of Medicine, “Carol Davila” University of Medicine and Pharmacy, 020021 Bucharest, Romania; daniela.ion@umfcd.ro; 3 3rd Medical Department, Psychiatry, The Faculty of Medicine, University of Medicine and Pharmacy “Grigore T. Popa”, University Street Nr. 10, 700115 Iași, Romania; alexandra_gaina@email.umfiasi.ro; 4 Department of Pathophysiology, National Institute for Infectious Diseases “Prof. Dr. Matei Balș“, 021105 Bucharest, Romania

**Keywords:** pediatric multiple sclerosis, Baló’s concentric sclerosis, cerebral MRI, tumefactive demyelinating lesion, MR spectroscopy

## Abstract

Background: Baló’s concentric sclerosis stands out as a rare form of multiple sclerosis that features large tumor-like demyelinating lesions, which resemble brain tumors and create significant diagnostic and therapeutic obstacles for pediatric patients. Case Presentations: We present two case studies of pediatric patients, aged 11 and 15, diagnosed with extensive cerebral tumefactive inflammatory lesions indicative of Balo’s type multiple sclerosis (MS). Both cases highlight the unique challenges faced in the diagnosis and treatment of this rare form of MS, characterized by the presence of large, tumor-like lesions that can mimic primary brain tumors. We will explore the diagnostic complexities, including the need for advanced imaging techniques, MR (Magnetic Resonance) spectroscopy, along with the time needed for differential diagnoses, which might delay the start of proper treatment. Current therapies, such as corticosteroids and immunomodulators, require customization to individual patients, carefully monitoring of clinical outcomes and possible side effects. This paper emphasizes that handling these cases requires a multidisciplinary approach, addressing not only the medical treatment but also the psychosocial needs of affected children and their families. By sharing these experiences, we aim to increase awareness about Balo’s type MS in pediatric populations and provide clinical insights into effective management strategies for similar cases in clinical practice. Conclusions: Timely detection of atypical demyelinating lesions together with immediate treatment intervention plays a crucial role in pediatric Baló-type MS. These cases demonstrate the essential role of advanced imaging and immunological testing in precise diagnosis while showcasing successful treatment approaches through corticosteroids and second-line immunotherapies, which improve patient outcomes in this atypical MS variant.

## 1. Introduction

Multiple sclerosis (MS) represents a chronic demyelinating inflammatory disease that targets the central nervous system (CNS) while following cyclical patterns of relapses and remissions. The prevalence of pediatric-onset MS constitutes less than 5% of all MS cases and shows an incidence rate of about 0.2–2.9% in individuals under 18 years old [1,2,3]. Children with MS often exhibit different clinical symptoms compared to adult MS patients. The tumefactive demyelinating lesions characteristic of Baló’s concentric sclerosis resemble other conditions like gliomas or infections, which lead to diagnostic delays [4,5]. Magnetic resonance imaging (MRI) brain scans show ring patterns, indicating the presence of concentric regions of demyelination and remyelination within white matter tissue, which characterizes the pathological nature of the disease [6,7]. Comprehensive spinal cord MRI at initial presentations in pediatric cases is strongly recommended by current guidelines to improve diagnostic certainty and exclude alternative central nervous system pathologies [8].

Baló’s disease was historically recognized as a fatal condition in most patients, according to previous studies [9,10,11]. Progress in imaging technology, together with new biochemical markers, has driven substantial advancements in the field. Recent observations demonstrate favorable results for numerous pediatric patients when modern imaging techniques are combined with aggressive treatment methods [12,13,14,15]. MR spectroscopy proves essential for distinguishing between tumefactive inflammatory lesions and neoplasms [16,17], which helps reduce the need for invasive brain biopsies during diagnosis [18]. The choice of MS treatments for children remains complicated because the number of approved medications is limited despite their proven effectiveness and because patient responses show marked variation [19]. Studies are ongoing to understand the long-term effects of multiple treatment approaches on children. This research paper discusses two pediatric MS cases that show distinct features of Baló’s concentric sclerosis. These cases demonstrate the diverse presentations of pediatric MS while also showing the crucial need for rapid recognition of uncommon symptoms, which can affect both diagnosis accuracy and treatment personalization for this rare disease.

## 2. First Case Report

A 15-year-old girl came to our clinic because she had been suffering from recurrent, severe headaches localized to the right temporal region for three months. The headaches first appeared as short bursts that happened three to four times weekly, with each episode lasting no more than 15 min. These episodes evolved into daily occurrences that lasted 3 to 4 h while frequently showing symptoms like sensitivity to light and sound, with nausea and vomiting. Before hospitalization, the patient experienced headaches that woke her up from sleep, which did not respond to nonsteroidal anti-inflammatory treatment. The patient had no recent history involving head trauma or serious infections. Her medical history showed no important medical events or health conditions and normal developmental progression. The patient’s family history showed no demyelinating diseases except for one sibling diagnosed with Turner syndrome, which doctors consider unrelated to the current medical situation.

At first, clinical and neurological examination yielded no abnormalities. Then, the patient displayed signs of intracranial hypertension, which led to the decision to perform a brain MRI. The brain imaging showed a significant tumefactive demyelinating lesion located in the right temporal lobe with dimensions of 23 × 19 × 21.5 mm.

**Figure 1:**
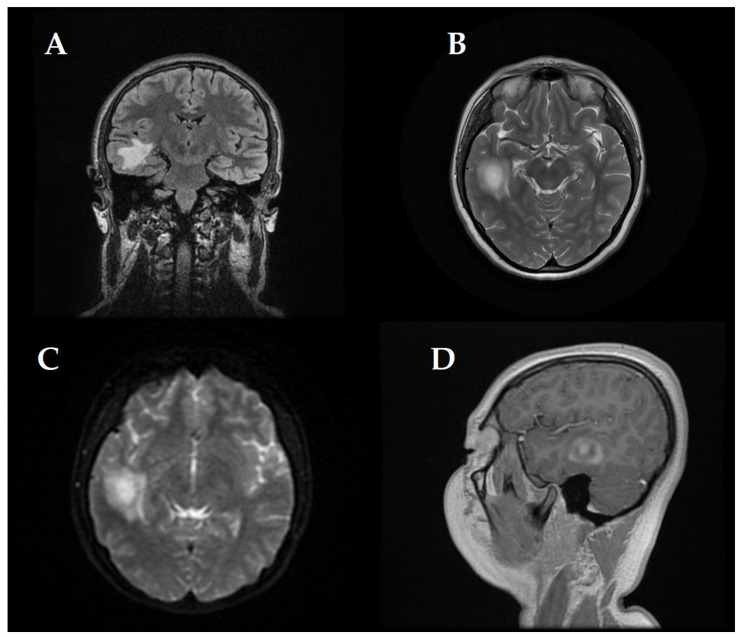
(**A**–**D**) First MRI shows (**A**) Coronal FLAIR sequence right temporal lesion, characterized by moderate peripheral vasogenic edema, Axial T2-weighted imaging (**B**) confirms the extent of edema around the lesion, (**C**) Diffusion-weighted imaging (DWI) reveals restricted water diffusion and Sagittal post-contrast T1-weighted imaging (**D**) highlights predominantly late peripheral contrast enhancement measuring 23 × 19 × 21.5 mm.

The lesion showed moderate perilesional vasogenic edema while demonstrating restricted diffusion and predominantly peripheral late gadolinium enhancement (Figure 1). The observed imaging pattern led the medical team to consider Baló’s concentric sclerosis-type lesion and glioma as potential diagnoses.

**Figure 2:**
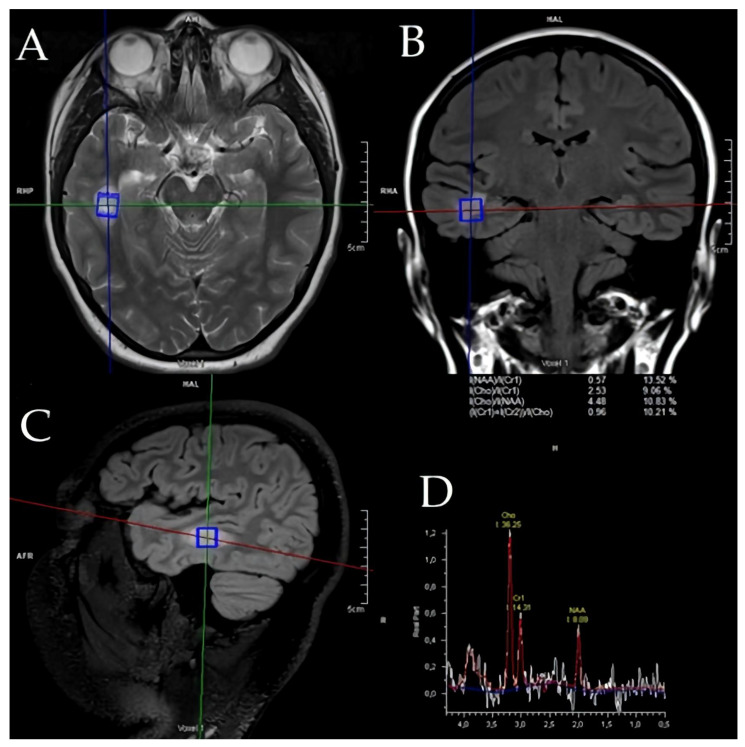
(**A**–**D**) One month later, (**A**) Axial T2-weighted MRI illustrating hyperintense lesion localization. (**B**) Coronal FLAIR sequence confirming lesion hyperintensity and delineating precise anatomical position. (**C**) Sagittal FLAIR sequence highlighting the lesion’s spatial distribution and surrounding tissue involvement. (**D**) MR spectroscopy profile displaying decreased N-acetylaspartate (NAA)/creatine (Cr1) ratio, elevated choline (Cho)/Cr1 ratio, and lactate peaks, indicating inflammatory demyelination rather than neoplastic tissue.

Another demyelinated region with similar features was present, and the measurement was less than one centimeter. The demyelinated area, which measured less than a centimeter, was identified as an “active plaque”. The lesion’s MR spectroscopy demonstrated a metabolic signature consistent with inflammatory demyelination instead of being a neoplastic tissue. The results can be viewed in Figure 2.

A spinal cord MRI encompassing cervical, thoracic, and lumbar regions was performed and revealed no lesions.

A full range of laboratory tests helped identify the underlying cause (refer to Table 1).

The CSF (Cerebrospinal Fluid) analysis revealed positive oligoclonal bands, which verified intrathecal immunoglobulin G production. The absence of MOG-IgG and AQP4-IgG in serological tests effectively excluded both neuromyelitis optica spectrum disorder and MOG-associated encephalomyelitis.

A significant elevation of anti-thyroid peroxidase (ATPO) antibodies was detected in the patient who received a confirmed diagnosis of autoimmune thyroiditis from endocrinological evaluation and subsequently received appropriate treatment. After a thorough review of the patient’s clinical information and laboratory tests, along with imaging scans, a diagnosis of Baló-type MS was established.

A high-dose methylprednisolone treatment of 1 g intravenously for 5 days was initiated, followed by a 2-week oral prednisone tapering regimen. The patient showed significant symptom improvement with reduced headache frequency and intensity and cessation of vomiting.

Follow-up MRIs taken at 6 months and then every year showed new demyelinating lesion appearance—cervical spinal lesions, while the original tumefactive lesion had reduced in size with no new contrast enhancement evident. As a result, the patient experienced an improved clinical condition since the headaches became less frequent and less severe; she stayed nearly symptom-free for six months, which resulted in no initiation of disease-modifying therapy. The patient received ongoing monitoring through clinical assessment and imaging surveillance instead of active treatment.

**Figure 3:**
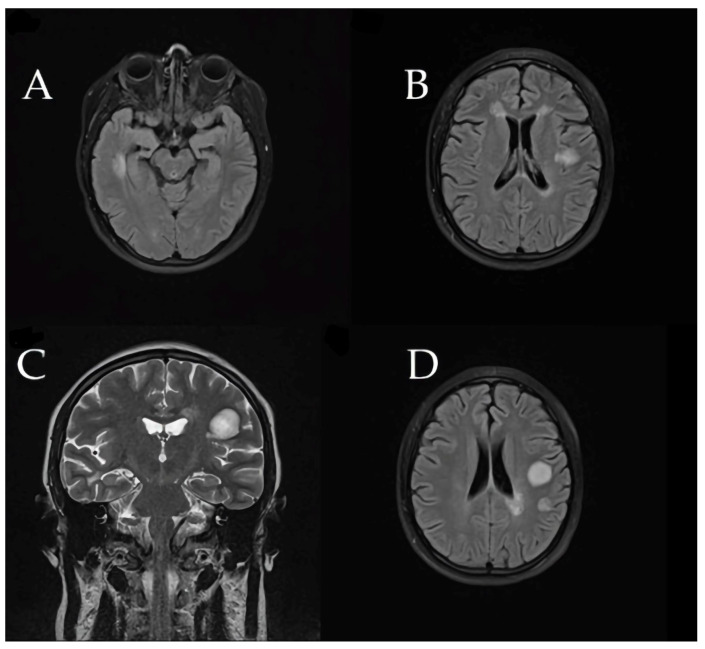
(**A**–**D**) Two years later, MRI reveals (**A**) Axial FLAIR sequence with marked regression in lesion size and surrounding edema compared to prior imaging. (**B**,**D**) Axial FLAIR sequences and (**C**) Coronal T2-weighted sequence identify multiple bilateral demyelinating lesions.

Two and a half years after the initial presentation, the patient developed new neurological symptoms like dysarthria along with right facial weakness and right-sided tongue deviation (see Figure 3).

The clinical presentation shows signs of right peripheral facial nerve palsy combined with hypoglossal nerve palsy. The patient received further treatment through intravenous methylprednisolone pulses before starting a gradual oral corticosteroid taper, which completely resolved the symptoms. The medical team recommended interferon-beta as a long-term treatment approach, but the family wished for a new therapy that was not approved for children, and initiation was delayed with spontaneous favorable evolution.

The EDSS score was systematically assessed after each relapse. Following both relapses occurring 2.5 years apart, the EDSS score returned to 0, indicating complete clinical recovery. However, an increase in EDSS (score of 2) was documented one month before the patient reached 18 years of age, which corresponded to attempts at tapering and withdrawing a low-dose oral corticosteroid therapy. Subsequently, distal limb paresthesia emerged, which worsened further following a COVID-19 infection. Corticosteroid therapy was reintroduced at a minimal dose, resulting in an improvement to an EDSS score of 1 over subsequent months. Cognitive function, assessed by the Montreal Cognitive Assessment (MoCA), remained consistently normal at both evaluations (scores of 29/30). Psychiatric assessments identified anxiety and mild depressive symptoms, which were managed effectively through intermittent medication, psychotherapy, and individual counseling. Although the patient exhibited good recovery following the second relapse event, with a notable reduction in lesion size, she remains dependent on corticosteroid therapy, emphasizing the critical need for prompt initiation of disease-modifying therapy (DMT).

This case demonstrates that early steroid intervention can have positive results for children with non-standard MS presentations, like Baló’s disease variants.

## 3. Second Case Report

An 11-year-old girl was evaluated after an episode of acute neurological symptoms consisting of blurred vision, dysarthria, and left-sided weakness. The symptoms began with transient visual impairment and occipital headache, which resolved after about four hours, but the left hemiparesis re-emerged later the same day and persisted. On examination, she had left central facial palsy and hemiparesis.

Brain MRI revealed a large tumefactive demyelinating lesion in the right frontal lobe (centrum semiovale region). Additionally, multiple smaller hyperintense lesions were observed in the white matter on T2/FLAIR images, raising a broad differential diagnosis that included inflammatory demyelination (atypical MS), CNS infection, or a vascular event (ischemia).

**Figure 4:**
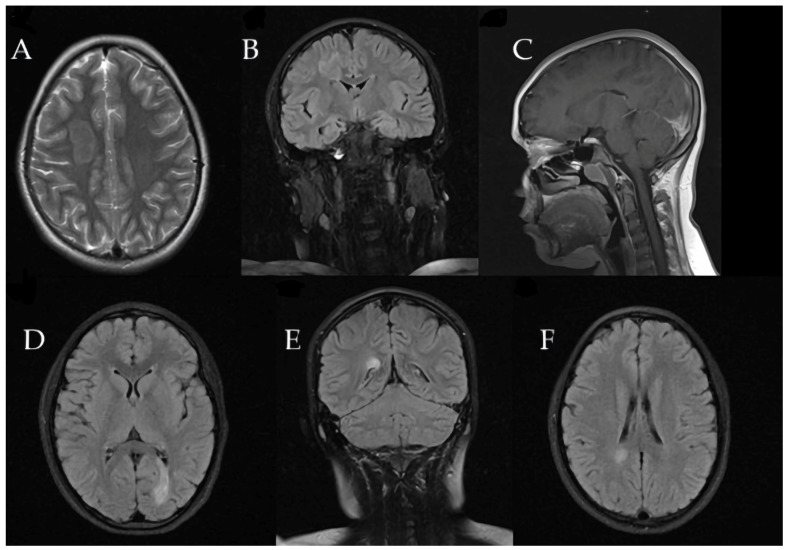
(**A**–**F**) First MRI shows right F-centrum semiovale lesion (**A**) Axial T2-weighted image reveals moderate peripheral vasogenic edema. (**B**,**D**–**F**) Coronal and axial FLAIR sequences clearly illustrate the hyperintense appearance of the lesion, perifocal edema, and bilateral demyelinating lesions. (**C**) Sagittal T1-weighted post-contrast sequence highlights late peripheral enhancement measuring 24 × 15.5 × 20.4 mm and lesion localization.

These initial imaging findings are shown in Figure 4.

A spinal cord MRI covering the cervical, thoracic, and lumbar regions was conducted and did not reveal any lesions.

A thorough diagnostic workup for infectious and autoimmune etiologies was performed (Table 2). Lumbar puncture demonstrated CSF oligoclonal bands, supporting a diagnosis of CNS demyelination. Blood tests for common viral agents revealed positive IgG titers (with negative IgM) for Epstein–Barr virus (EBV), indicating a past infection; no evidence of active infection (e.g., other viral PCRs or cultures) was found. The serum was negative for MOG and AQP4 antibodies, and other autoimmune markers, including antinuclear antibodies, were unremarkable. The overall profile suggested an immune-mediated demyelinating process rather than infection.

The patient was treated with intravenous methylprednisolone (1 g/day for 5 days), followed by a 2-week oral prednisone taper. This treatment led to the complete remission of her acute neurological deficits. Visual evoked potentials were also obtained and were within normal limits, providing no evidence of optic nerve involvement at that time. A psychiatric evaluation identified adjustment difficulties with depressive features in the patient and high stress within the family; psychological support and counseling were initiated.

**Figure 5:**
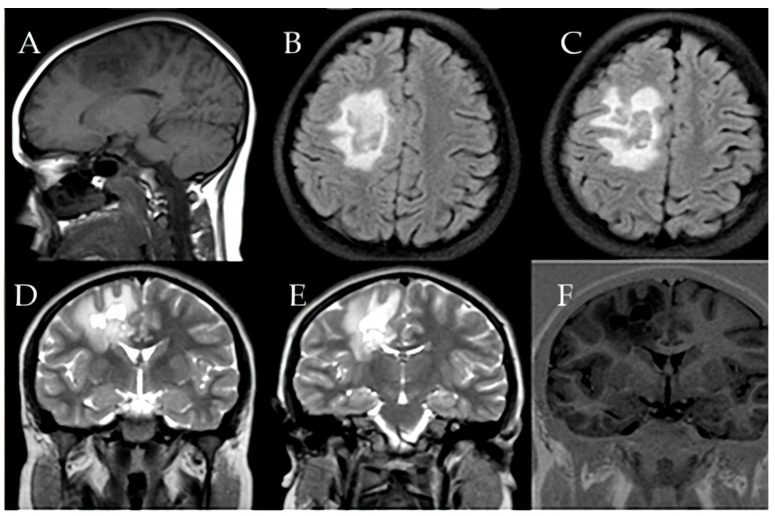
Three months later, right frontal lesion increased to 38 × 25 × 23.3 mm with impressive surrounding edema. (**A**) Sagittal T1-weighted image illustrating lesion extent in the right frontal lobe. (**B**,**C**) Axial FLAIR images showing hyperintense lesion characteristics and perifocal edema. (**D**,**E**) Coronal T2-weighted image depicting lesion enlargement and extensive surrounding edema in the affected area. (**F**) Coronal T1-weighted MRI sequence without contrast demonstrates persistent perifocal edema around the previously identified lesion, with no evidence of new lesion formation.

Three months later, the patient developed recurrent paravertebral muscle twitches. MRI at that time showed persistent, significant edema around the prior lesion, although no new lesions were detected (Figure 5).

A neurosurgical consultation was obtained due to the tumefactive nature of the lesion; the neurosurgeon recommended continued clinical and radiologic surveillance and deferred brain biopsy, given the improving trend and lack of new findings.

**Figure 6:**
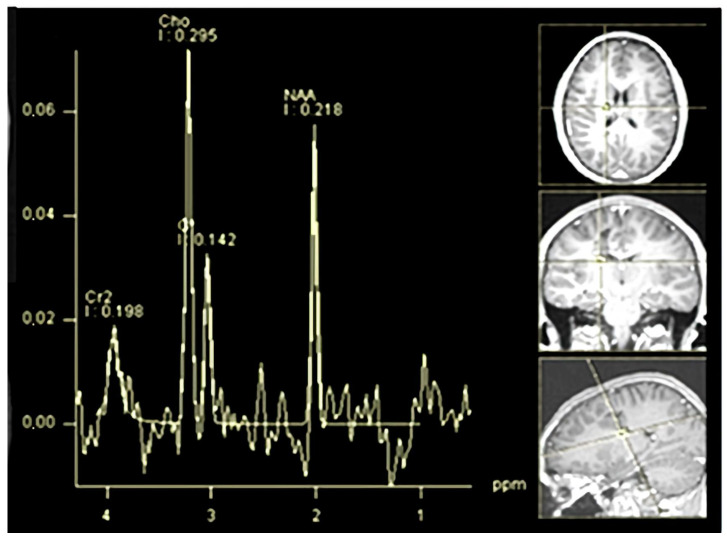
Three months later, spectroscopy highlights increased cho/cr1 ratio and peaks of lactate.

An MR spectroscopy study of the lesion (Figure 6) was consistent with an inflammatory demyelinating pathology, further supporting the diagnosis of Baló’s type MS. Over this period, the patient remained in a stable condition without new relapses.

**Figure 7:**
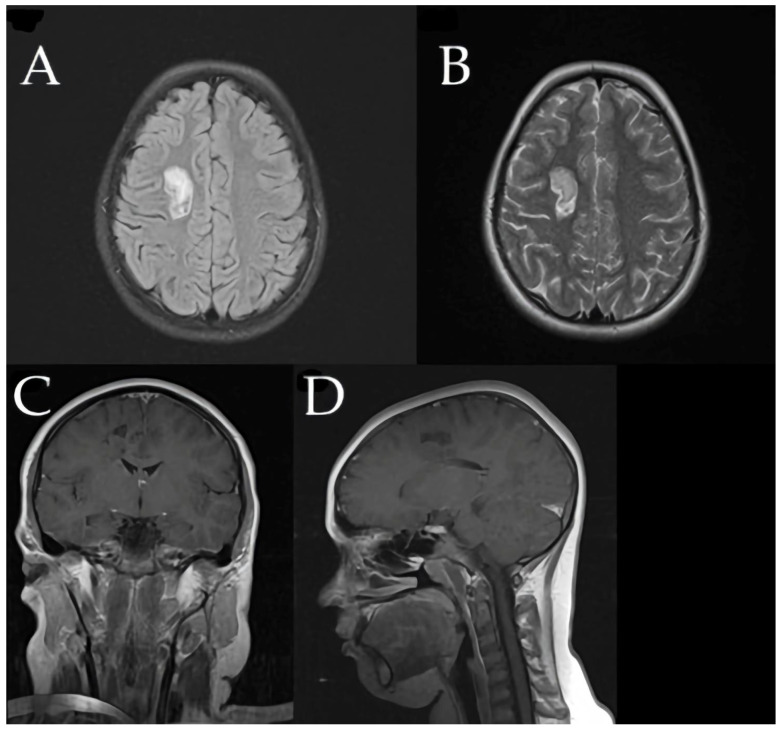
(**A**–**D**) One year later, MRI revealed significant decrease in concentric sclerosis 18 × 14 × 12.2 mm, with no new demyelinating lesions. Axial FLAIR (**A**) and axial T2-weighted (**B**) images demonstrate reduced lesion size and diminished hyperintense signal intensity. Post-contrast coronal (**C**) and sagittal (**D**) T1-weighted images confirm absence of active inflammation or enhancement, highlighting lesion stability and significant regression from initial presentation.

Subsequent MRIs showed a gradual reduction in the size of the demyelinating lesions (Figure 7).

The second demyelinating attack occurred 3 years and 6 months after the initial presentation, manifesting again as left-sided hemiparesis. A third relapse followed 9 months later with similar symptoms. Each relapse was promptly treated with high-dose corticosteroids, resulting in full symptom resolution. After the third episode, the family opted for a B-cell-depleting therapy instead of interferon-beta (which was the standard immunomodulatory option available in our country for pediatric MS at that time). The patient received rituximab infusions abroad. This treatment yielded a favorable outcome: in the year following rituximab, she experienced no further clinical relapses, and follow-up MRI showed no new lesions, while the existing concentric lesion had substantially reduced in size.

The EDSS score was also consistently evaluated following each relapse event. Each evaluation indicated full neurological recovery (EDSS = 0), with the final assessment before the patient reached 18 years of age also maintaining an EDSS of 0. The patient received physiotherapy during both relapses and recovery periods, which, together with medical treatment, helped to speed up and fully restore functionality.

Cognitive functioning assessed approximately 18 months after the initial onset (at 13 years of age) was within normal ranges according to standardized neuropsychological evaluations (WISC IV: IQ = 126), although some minor deficits were noted in processing speed. Performance on the Symbol Digit Modalities Test (SDMT) and Child Behavior Checklist version 2 (CBC2) was normal. Psychiatric evaluations performed further documented depressive episodes, predominantly during the year, with two relapses. These were effectively managed using intermittent medication and psychotherapy, transitioning to supportive counseling alone during the final two years.

This case illustrates the diagnostic and therapeutic challenges of Baló’s type MS in a child, as the condition can closely mimic other neurological diseases, requires an adaptable treatment strategy, and also emphasizes the critical importance of early initiation of disease-modifying therapy to achieve favorable long-term outcomes.

## 4. Discussion

Baló’s concentric sclerosis (BCS) is an atypical variant of MS, and its presentation in pediatric patients can be particularly challenging. When MRI brain scans show concentric ringed lesions, clinicians must consider this diagnosis, even in pediatric patients. Patients with this condition show severe focal neurological deficits and even cognitive or behavioral changes, and these symptoms make both the diagnostic process and the therapeutic management difficult [13,20,21]. The combination of early age onset and atypical imaging findings requires careful consideration because they share characteristics with several neurological disorders, such as acute disseminated encephalomyelitis or tumors, demanding a high index of suspicion and a multidisciplinary approach (see Table 3).

Using advanced imaging techniques like MR spectroscopy and diffusion imaging can be crucial in revealing an inflammatory demyelinating process in these large lesions, which helps clinicians avoid unnecessary invasive procedures. In our diagnostic process, MRI scans were performed on a 1.5 Tesla and followed the imaging protocols established by experts from the International Society for Multiple Sclerosis [18,22,23,24]. The MR spectroscopy protocol employed included acquisitions at both short (TE = 30 ms) and intermediate (TE = 135 ms) echo times. Spinal cord MRI (encompassing cervical, thoracic, and lumbar regions) was also performed for both patients. This practice is in line with pediatric MS guidelines [8], which strongly recommend imaging the entire spinal cord in children with a first demyelinating event to identify additional lesions. However, these evaluations were not performed immediately at disease onset but within 1 to 3 months following the initial cerebral MRI. The initial spinal MRIs were normal for both patients. Subsequent spinal imaging included only cervical spine MRIs, conducted approximately every 1 to 2 years and not necessarily at the same time as each cerebral MRI. Over time, cervical lesions were identified in the first patient. Prior to reaching 18 years of age, the first patient underwent cerebral and spinal screening within our clinic, whereas the second patient had a cerebral evaluation at our clinic and probably a complete one abroad.

Both pediatric patients included in this study presented with supratentorial tumefactive lesions, which represent a more common lesion localization in BCS, as indicated in the literature [13,24]. Both cases exhibited favorable responses to high-dose corticosteroid therapy during relapses, consistent with its recognition as first-line therapy in most scenarios. Additionally, when patients demonstrate inadequate response to corticosteroids, plasmapheresis constitutes a viable therapeutic option [10,13].

The two cases described highlight different trajectories within the spectrum of Baló’s type MS. The first case demonstrated a relatively monophasic course during the early years, with long symptom-free periods and cerebral MRI evidence of lesion regression under corticosteroid treatment alone. In contrast, the second case had numerous relapses, which led to the implementation of second-line immunotherapy (rituximab). These observed differences reflect what has been reported in the literature—BCS in some pediatric cases can remain isolated or have a benign course, whereas others evolve into a more standard relapsing-remitting MS pattern [15,16].

Both patients benefited from acute corticosteroid therapy, which is the first-line treatment for tumefactive MS lesions and often leads to rapid clinical and radiological improvement. However, given the aggressiveness of BCS lesions, clinicians must remain vigilant since some patients need supplementary immunomodulatory treatments. In our second case, the decision to use rituximab was driven by recurrent relapses and the family’s preference to avoid interferon.

Until 2022, interferon-β was the only approved disease-modifying treatment available in Romania for pediatric-onset MS. Fingolimod became accessible only after this date, providing a valuable additional treatment option. Both cases discussed here were initially diagnosed and managed before the availability of Fingolimod. In alignment with international guidelines recommending early treatment initiation for aggressive pediatric MS cases [10,13,25], interferon-β was proposed to both families quite soon after diagnosis. However, both families opted to delay this medication due to concerns about its mode of administration and its moderate efficacy profile, despite interferon historically serving as the standard first-line treatment [25].

For Case 1, the disease followed a relatively mild natural course initially, with the second relapse occurring 2.5 years after disease onset and effectively managed with high-dose corticosteroids. Following the second relapse, the patient, approaching 18 years of age, again postponed interferon treatment, anticipating access to newer therapeutic options upon reaching adulthood.

In Case 2, approximately three years separated the first two relapses, and a similar situation occurred regarding interferon-β initiation as in the previous case. However, after the third relapse, which was closer to the second, the family became increasingly concerned about further relapses occurring. They decided to seek treatment abroad, and rituximab was proposed and started there.

Fortunately, both patients experienced favorable outcomes, highlighting the importance and impact of patient and family preferences in clinical decision-making.

We advocate for adding disease-modifying therapies in managing BCS to improve prognosis, particularly in patients (like patient 2 in our case report) whose clinical and radiological presentations closely align with classical MS phenotypes. Rituximab is specifically recommended based on its demonstrated therapeutic efficacy in such cases [25,26,27].

Recent research highlights a significant shift in understanding MS pathogenesis, emphasizing the critical role of B cells in addition to T cells. Evidence increasingly supports a B cell–T cell interactive model rather than the previously T-cell-driven mechanism. Peripheral B cells exert pro-inflammatory actions, and their depletion can effectively reduce pro-inflammatory responses mediated by CD4+ and CD8+ T cells [25]. However, B cells also have anti-inflammatory properties, complicating their overall role. By depleting CD20-positive B cells without affecting B cells residing within lymphoid tissues, Rituximab reduces inflammation and demyelination, contributing to better clinical outcomes by decreasing both the number of new central nervous system lesions and relapse frequency, which explains its effectiveness in our second case. Nevertheless, the long-term safety profile of rituximab remains incompletely understood, highlighting the importance of ongoing vigilance regarding potential risks [25].

Given the rarity and clinical heterogeneity of BCS, reflected in predominantly isolated case reports or small case series, there is currently no standardized therapeutic protocol. Nevertheless, there is broad consensus favoring early initiation of high-efficacy treatment in managing atypical and aggressive demyelinating conditions such as BCS.

These cases emphasize the importance of multidisciplinary care approaches. Both patients and their families faced substantial stress and psychological suffering because of uncertain diagnoses and the intensity of treatments. The addition of mental health support along with rehabilitation services became essential to the complete care plan for pediatric MS to ensure both patients and their families received appropriate support. Early involvement of specialists (neurologists, neuroradiologists, neurosurgeons, psychiatrists, and physiotherapists) ensured that each aspect of the patient’s condition was managed appropriately. For instance, the neurosurgical treatment in Case 2 prevented an unnecessary biopsy, while psychiatric expertise in both cases helped manage anxiety and mood symptoms related to chronic illness.

The primary limitation of our study is the notably small sample size, consisting of only two cases, which significantly restricts the generalizability of treatment outcomes and disease progression observed. Nonetheless, we believe that these case examples may provide practical insights for clinicians managing similar rare pediatric MS presentations. We strongly advocate for future multicenter cohort studies involving larger patient populations to more accurately delineate clinical features and establish evidence-based, targeted therapeutic approaches.

To better distinguish treatment effects from the natural disease progression or placebo effects on neurological outcomes, future studies involving very small samples may benefit from incorporating historical controls or adopting single-case experimental designs (N-of-1 trials).

Overall, the presented cases demonstrate the importance of including Baló’s concentric sclerosis in the differential diagnosis of large demyelinating brain lesions, although it is rare among pediatric patients. Timely administration of corticosteroids produces significant therapeutic advantages. Differences in patient responses to initial treatment highlight the need for personalized long-term management: some patients may do well with careful observation after initial treatment, whereas others might require ongoing immunotherapy to prevent further relapses. A combination of future studies and additional case reports will help us better understand the pathophysiology and treatment approaches for this uncommon type of MS in young patients.

## 5. Conclusions

The presence of atypical tumefactive demyelinating brain lesions in pediatric patients seen through MRI examinations should lead clinicians to evaluate Baló’s concentric sclerosis as a potential diagnosis. Accurate diagnosis of these lesions requires early use of advanced imaging techniques together with immunological assays to distinguish them from tumor or infectious mimickers. Patients treated with high-dose corticosteroids show rapid improvements in clinical symptoms and MRI findings. Second-line treatments such as rituximab demonstrate effectiveness in recurrent disease cases, as shown here. The published pediatric case experiences similar to those in this research study must be shared widely to improve clinical practices for this unusual MS variant. The different presentation of pediatric Baló’s disease cases highlights the existing knowledge gaps of this condition while stressing the need for ongoing documentation and research to create improved diagnostic methods and treatment strategies.

## Figures and Tables

**Table 1:** Laboratory results from blood and CSF for Case 1 at onset and 1 year later.

Investigations	Blood at Onset	CSF at Onset	Blood at 1 Year	CSF at 1 Year
Usual hematology and Biochemistry	Normal	Norma	Normal	Not performed
25 OH vit D	Low	Not performed	Normal	Not performed
Infectious screening	* Negative	** Negative	Not performed	Not performed
Immunological screening ***	Negative	Not performed	Not performed	Not performed
Thyroid screening	High ATPO (310 UI/mL)	Not performed	High ATPO (264 UI/mL)	Not performed
Anti-AQP4, anti-MOG	Negative	Not performed	Negative	Not performed
Index IgG	Negative	Negative	Not performed	Not performed
Oligoclonal Bands	Negative	Positive	Not performed	Not performed

* Infectious screening—C-reactive protein, ferritin, erythrocyte sedimentation rate, HIV, TPHA, HAV, HCV, EBV, CMV, HSV, mycoplasma, Borrelia. ** Infectious screening—Borrelia, measles. *** Immunological screening (especially for SLE): ANA, RF, CIC, C3, C4, anti-double-stranded DNA antibodies, anti-neutrophil cytoplasmic antibodies; thyroid screening: TSH T3, fT4, anti-TPO.

**Table 2:** Key cerebrospinal fluid and serological investigation results for Case 2.

Investigations	Blood at Onset	CSF at Onset	Blood at 3 Years	CSF at 3 Years
Usual hematology and Biochemistry	Normal	Normal	Normal	Normal
25 OH vit D	Low	Not performed	Normal	Not performed
Infectious screening	* IgG EBV positive	** Negative	Not performed	Not performed
Immunological screening ***	Negative	Not performed	Not performed	Not performed
Thyroid screening	Negative	Not performed	Not performed	Not performed
Anti-AQP4, anti-MOG	Negative	Not performed	Negative	Not performed
Index IgG	Negative	Negative	Negative	Negative
Oligoclonal Bands	Negative	Positive	Negative	Positive

* Infectious screening—C-reactive protein, ferritin, erythrocyte sedimentation rate, HIV, TPHA, HAV, HCV, EBV, CMV, HSV, mycoplasma, Borrelia ** Infectious screening—Borrelia, measles. *** Immunological screening (especially for SLE): ANA, RF, CIC, C3, C4, anti-double-stranded DNA antibodies, anti-neutrophil cytoplasmic antibodies; thyroid screening: TSH, T3, fT4, anti-TPO.

**Table 3:** Differential Diagnosis for Pediatric Patients with Tumefactive Demyelinating Lesions.

Differential Diagnosis	Key Diagnostic Considerations
Baló’s concentric sclerosis	MRI showing concentric demyelination, MR spectroscopy indicating inflammatory demyelination
Primary brain tumor (glioma)	MRI lesion characteristics, MR spectroscopy indicating neoplastic changes, histopathological confirmation needed
Acute disseminated encephalomyelitis (ADEM)	Multifocal lesions, rapid onset, typically post-infectious or post-vaccination
Neuromyelitis optica spectrum disorders (NMOSD)	Presence of AQP4 **-IgG antibodies, specific MRI spinal cord lesions, optic nerve involvement
MOG *-associated encephalomyelitis	Presence of MOG-IgG antibodies, optic neuritis, spinal cord involvement
Infectious etiologies (e.g., abscess)	Fever, systemic symptoms, specific infectious markers, positive cultures or PCR tests

* MOG—Myelin Oligodendrocyte Glycoprotein. ** AQ4—Aquaporin 4 antibodies.

## Data Availability

All relevant data supporting the findings of this case report are contained within this manuscript. Additional details can be obtained from the corresponding author upon reasonable request.

## Abbreviations

The following abbreviations are used in this manuscript:

MS	Multiple Sclerosis
MRI	Magnetic Resonance Imaging
CNS	Central Nervous System
CSF	Cerebrospinal Fluid
EBV	Epstein Barr Virus
MOG-IgG	Myelin oligodendrocyte glycoprotein antibody
AQP4-IgG	Aquaporin-4 antibodies
ATPO	Anti-Thyroid Peroxidase
FLAIR	Fluid Attenuated Inversion Recovery
PCR	Polymerase Chain Reaction
BCS	Baló’s Concentric Sclerosis
EDSS	The Expanded Disability Status Scale
COVID-19	Coronavirus Disease 2019
MoCA	Montreal Cognitive Assessment
DMT	Disease-Modifying Therapy
WISC	Wechsler Intelligence Scale for Children
SDMT	Symbol Digit Modalities Test
CBC2	Child Behavior Checklist version 2
NMOSD	Neuromyelitis optica spectrum disorders
ADEM	Acute disseminated encephalomyelitis
TE	Echo Time
